# Comparable rates of lumbar disc degeneration at long-term following adolescent idiopathic scoliosis spinal fusion extended to L3 or L4: systematic review and meta-analysis

**DOI:** 10.1007/s43390-024-00849-4

**Published:** 2024-03-28

**Authors:** Alberto Ruffilli, Matteo Traversari, Marco Manzetti, Giovanni Viroli, Elena Artioli, Simone Ottavio Zielli, Antonio Mazzotti, Cesare Faldini

**Affiliations:** IRCCS Istituto Ortopedico Rizzoli, 1st Orthopaedics and Traumatology clinic –University of Bologna, Bologna, Italy

**Keywords:** Adolescent idiopathic scoliosis, Spinal fusion, LIV, Degenerative disc disease, Adjacent segment disease, Pedicle screw

## Abstract

**Purpose:**

Surgical treatment of adolescent idiopathic scoliosis (AIS) requires a careful choice of fusion levels. The usual recommendation for the selection of the lowest instrumented vertebra (LIV) for double major or thoracolumbar/lumbar (TL/L) curves falls on L3 or L4. The aim of the present study is to assess if the spinal fusion with LIV selection of L3 or L4 in AIS patients has a clinical or radiological impact in terms of degenerative disc disease (DDD) in distal unfused segments at long-term follow-up.

**Methods:**

A systematic search of electronic databases from eligible articles was conducted. Only studies regarding long-term follow-up of AIS patients treated with spinal fusion were included. Clinical and radiographic outcomes were extracted and summarized. Meta-analysis on long-term follow-up MRI studies was performed. *p* value < 0.05 was considered significant.

**Results:**

Fourteen studies were included, for a total of 1264 patients. Clinical assessment of included patients showed a slight tendency to have worse clinical outcomes if spinal fusion is extended to L4 rather than L3. Despite that, meta-analysis could not be performed on clinical parameters because of heterogeneity of evaluated PROMs in included studies. Magnetic resonance imaging (MRI) evaluation at long-term follow-up showed no significant difference in terms of disc degeneration rate at overall meta-analysis (*p* = 0.916) between patients fused to L3 and L4.

**Conclusion:**

The LIV selection of L3 rather than L4, according to current literature, does not prevent disc degeneration in distal unfused segments over the long term. Long-term studies of patients treated with contemporary spinal instrumentation are needed.

**Supplementary Information:**

The online version contains supplementary material available at 10.1007/s43390-024-00849-4.

## Introduction

Adolescent Idiopathic Scoliosis (AIS) is a three-dimensional spinal deformity that affects 2–3% of the teenage population [1, 2]. Surgical correction for AIS is advised for large and progressive curves as prophylactic treatment to prevent further complications to cardiovascular and respiratory systems, and for aesthetic purposes.

The surgical treatment of AIS requires a careful choice of fusion levels. The spinal fusion will involve sacrificing motion segments, making the selection of fusion levels more critical when sacrificing highly mobile levels, such as those in the lumbar spine, since the fused spine tends to move as a single unit determining increased stress in unfused regions and eventually leading to early degenerative changes. Several factors, including an imbalanced spine in both the coronal and sagittal planes as well as a tilted lowest instrumented vertebra (LIV), have been previously linked to disc degeneration (DD).

The goals of AIS surgery are to minimize risk of future deformity progression, to optimize spinal balance both in the coronal and sagittal plane and fuse the least possible number of motion levels [3].

Historically, the fusion area was considered to be the portion of the spine between two stable vertebrae [4], and this was mainly due to the limitations of available instrumentation, such as the Harrington instrumentation, which allowed correction solely based on distraction forces. The advent of segmental instrumentation allowed spinal surgeons to apply translation and derotation forces on all instrumented levels; therefore, it has become possible to preserve motion levels distally [5]. Up to date, the selection of lowest instrumented vertebra (LIV) when fusing a TL/L curve is substantially limited to L3 or L4, in rare cases L5 [3, 5–11].

Current criteria for choosing L3 rather than L4 as the LIV in TL/L curves are as follows:

- L3 reaches the center sacral vertical line (CSVL) on bending radiographs towards the concavity of the lumbar curve.
- L3 exhibits vertebral rotation according to Nash–Moe < 2 on bending radiographs towards the convexity of the lumbar curve.
- L3–L4 disc wedging becomes neutral in concave side-bending radiograph.
- L4 vertebra has a coronal tilt < 20°.

**Fig. 1:**
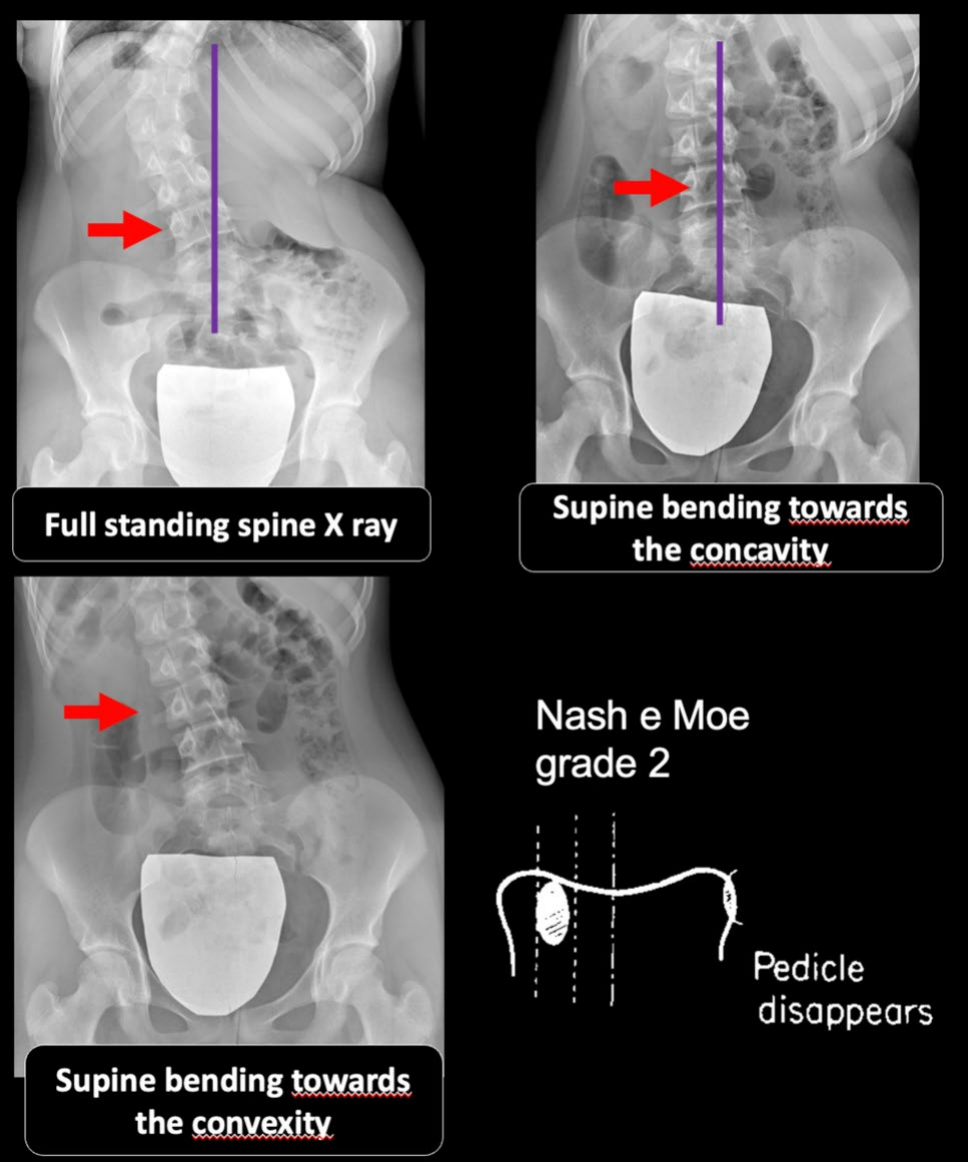
Criteria for LIV selection of L3 based on supine bending X-rays in TL/L curves according to Kim et al. and Shao et al.

However, even with clear criteria of LIV selection (Fig. 1), there is still a certain a lack of agreement among surgeons regarding the choice of LIV due to variability in bending radiographs and projection errors or artifacts. Some authors suggest conducting full spine supine bending radiographs under orthopedic consultant supervision[12].

The consequences of the preservation of one unfused distal segment below a long spinal fusion in an adolescent patient are still subject to debate. Outcomes have been investigated in few research papers, but a consensus has not yet been reached.

The aim of the present paper is to systematically review the current literature regarding long-term radiographical and clinical outcomes of adolescent idiopathic scoliosis surgery in which spinal fusion has been extended in the lumbar spine, to provide additional guidance to spine surgeons when choosing between fusion to L3 and L4.

## Materials and methods

### Review design

A systematic review of the literature regarding long-term outcomes of adolescent idiopathic scoliosis surgery was carried out following the Preferred Reporting Items for Systematic Reviews and Meta-Analyses (PRISMA guidelines) [13].

The Oxford level of evidence scale [14] was used to assess the level of evidence of the included studies (full version for randomized and non-randomized clinical trials, modified version for all other studies).

Inclusion criteria: papers describing the radiological and clinical outcomes of adolescent idiopathic scoliosis surgery with minimum of 5 years of follow-up.

Exclusion criteria: incomplete data regarding clinical outcomes measurements, LIV selection, and radiological data. Isolated case reports/series with less than 5 patients. In vitro studies and animal model studies.

Only articles in English on peer-reviewed journals who met the population, intervention, comparison, and outcomes criteria on systematic reviews were considered for inclusion.

Randomized-controlled trials, prospective and retrospective cohort studies, and case series (CS) were considered for inclusion.

### Search strategy

Pubmed-MEDLINE, The Cochrane Central Registry of Controlled Trials, Google Scholar, and the Embase Biomedical Database were searched over the years 1980–2023 to identify eligible studies describing the clinical and radiological outcomes in surgically treated adolescent idiopathic scoliosis patients at long-term follow-up. The online literature search was conducted in June 2023 by two authors (MM, and MT). The authors stated the following research questions: “*Are there radiological and clinical differences in long-term surgically treated adolescent idiopathic scoliosis patients in whom L3 or L4 were selected as LIV? Does LIV selection of L3 prevents from disc degeneration?*”.

The search was conducted using combinations of the following keywords: “scoliosis”, “MRI”, “long-term outcomes”, “PROMs”, “degenerative disc disease”, “DDD”, “adjacent segment degeneration”, “ASD”. Details on the search strategy are summarized in Supplementary Table S1.

### Study selection

**Fig. 2:**
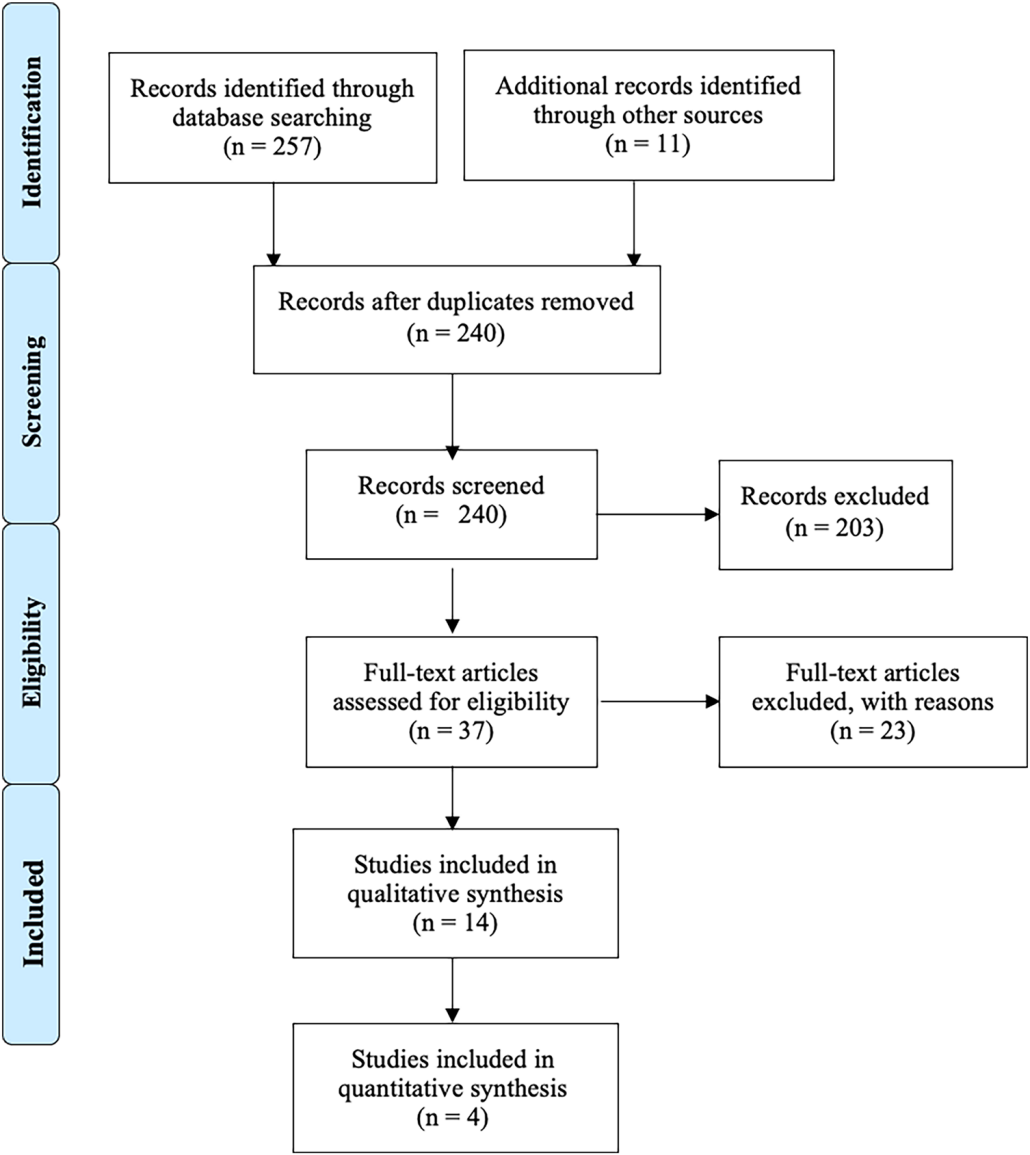
PRISMA flow diagram and the selection of studies

After screening the titles and abstracts, the full-text articles were obtained and reviewed. A manual search of the bibliography of each of the relevant articles was also performed to identify potentially missed eligible papers. Reviews and meta‐analyses were also analyzed to potentially broaden the search for studies that might have been missed through the electronic search. Duplicates were removed. The study selection process was carried out in accordance with the PRISMA flowchart [13] (Fig. 2). The present systematic review was accepted for registration in the PROSPERO database for systematic reviews [15] (ID: CRD42023438172).

### Data extraction

Two authors (MM and MT) extracted the data through a standardized data collection form. Three authors (MM, FB, and MT) checked the data for accuracy, and inconsistent results were discussed. Data concerning study design, number of patients, demographics of patients, Lenke type or curve pattern, LIV selection, follow-up time, clinical assessment, radiological assessment, and results were extracted and summarized in Table 1. The following outcomes were considered for analysis*:* presence of low back pain; PROMs scores; Pfirmann [16] and Modic [17] grading, coronal and sagittal alignment, and LIV tilt.

**Table 1:** Characteristics of treated patients, surgical treatments, evaluated outcomes and main findings of included studies;

Author (Year)	Study design	N° Patients (M:F)	Age at surgery (years)	Deformity pattern (Lenke Type\curve location)	Curves fused to L3 or L4 characteristics	Spinal instrumentation	LIV	LIV selection criteria	Mean Follow-up	Outcomes	Radiological parameter	Clinical Parameters	Results
Hayes (1988)	Retrospective case series	48 AIS patients (3:45) 59 Controls	26 (range, 12–38)	NR	NR	Harrington rod	From L1 to S1	NR	11 years (range, 1–23)	Presence of low back pain, X-ray evaluation (lateral maximum flexion and extension view of lumbar spine and standing lumbar spine view)	X-ray: translational motion according to Depuy method, disch height, retrolisthesis, lumbar lordosis	Moskovitz, Moe, Winter and Binner low back pain classification	Highest % of LBP in patients with LIV L4 Patients fused to L4 will have an increased incidence of retrolisthesis, and increased translational motion when compared with patients fused to higher levels
Fabry (1989)	Retrospective case series	182 (44:138)	14 (10–20)	82 single curves • 38 dextro-convex thoracic • 4 sinistro-convex thoracic • 28 dextro-convex thoracolumbar • 12 sinistro-convex thoraco-lumbar 100 double curves • 4 thoracic • 96 thoracolumbar	NR	Harrington rod	L1: 16 L2: 54 L3: 34 L4: 32 L5: 26 S1:4	In patients with double-major thoracolumbar curve the fusion area was extended below L3 All single major thoracolumbar curves were fused to L2	7 years (range, 4–14)	Presence of low back pain	NR	Arbitrary Scale of Back pain and activity Grading	Presence of LBP increased from L1 to L5
Krismer (1993)	Retrospective case series	49 (7:42)	17 (11–22)	NR	NR	Harrington rod In six patients ventral derotation and fusion or Dwyer anterior fusion was carried out as first surgical time	T12\L1: 12 L2: 17 L3: 6 L4: 9 L5: 5	NR	14 years (range 11–22)	X-ray evalutation, pain questionnaires	Anteroposterior + lateral full spine X-ray and flexion extension lumbar spine x: Cobb angle, lumbar lordosis, anteroposterior translation	Dallas pain questionnaire, Ransford pain drawings	Pain occurred in 80% of patients with LIV $\ge 4$, but no relationship between fusion level and pain or radiological parameter was found
Perez-Grueso (2000)	Retrospective case series	35 (2:33)	15 (11 ± 8—25 ± 5)	26 double curves 2 single curves 6 thoraco-lumbar 1 single thoracic	NR	Harrington rod	L3: 15 L4: 18 L5: 2	Cotrel-Dubousset criteria	10 years	X-ray evaluation, QoL questionnaire, MRi evaluation	Mri = 1) diminished disc height (least 50% compared with that in the superior or the inferior level when the evaluated disc was the one directly below the instrumentation) 2) Signal Intensity 3) Presence of bulging, protrusion or extrusion X- ray: Cobb angle, apical vertebral translation, C7PL, CSVL, thoracic kyphosis, thoraco-lumbar lordosis, lumbar lordosis, SVA, segmental motion	SRS clinical outcome questionnaire + Moskowitz lumbar pain scale	There was no correlation between the length of fusion, the number of free lumbar segments, radiographic results, MRI findings, dynamic results and the final clinical outcome A higher rate of anteroposterior translation of < 3 mm was found between LIV L3 and L4 (2\13 vs 6\30; 15% vs 20%, respectively)
Danielsson (2003)	Retrospective case–control study	139 AIS patients (10:129) 100 controls (10:90)	15 (1.8)	Double primary: 32 Thoracic: 94 Thoraco-lumbar: 11 Lumbar:2	NR	Harrington rod	T12: 7 L1,2 and 3: 95 L4,5: 37	NR	23 yrs	QoL questionnaires, X-ray evaluation (included disc degeneration sign)	Full spine X-ray: Cobb angle, thoracic kyphosis, lumbar lordosis Disc degeneration: Measured at X-ray in terms of space narrowing	Visual analogue scale, pain drawings, McGill Pain questionnaire, SF-36, Oswestry low back pain questionnaire	No significant differences in disc degeneration between patients fused to L3 or higher versus L4 or lower. No significant differences in terms of LBP between patients to L3 versus L4
Bartie (2009)	Retrospective case–control study	171 AIS patients (18:153) vs 209 healthy controls	18	NR	NR	Harrington rod	L2: 59 L3: 52 L4: 143 L5: 12	NR	10 years	QoL questionnaire, X-ray radiographic evaluation (only 88 patients)	Full spine X-ray: curve pattern, cobb angle, status of instrumentation, presence of pseudoarthrosis, LIV tilt, C7 coronal plumbline, cervical lordosis, thoracic kyphosis, total lumbar lordosis, lowest fused segment to S1 lordosis Disc degeneration: Disc space heights in the unfused area were measured on the preoperative, postoperative follow ups, and flexion/ extension radiographs. Ratios were established from these measurements by comparison to the dimensions of the L4 vertebral body on each radiograph	Presence of back pain and its characteristics (duration, frequency, severity), SF-36	No statistically significant differences in terms of pain or radiographic parameters between L2\L3\L4 groups but L4 had a slightly higher LBP intensity
Nohara (2015)	Retrospective cohort study	93 (3:90)	15.2 (11–20)	Lenke 1: 58 Lenke 2: 23 Lenke 3: 4 Lenke 4: 0 Lenke 5: 3 Lenke 6: 5	NR	Hybrid instrumentation (screws, hooks, wires)	T11 + T12: 11 L1: 29 L2: 23 L3: 28 L4: 2	Lenke 1A were fused at stable vertebra or one level above based one supine-bending x-rays	12,8 years (10–17)	MRi evaluation, X-ray evaluation, presence of LBP	X-ray: Cobb angle, wedging angle of each vertebra of fused segments, tilt angle, maximum tilt of unfused segments, LIV sacrum, and the coronal balance Sagittal balance, Thoracic kyphosis, lumbar lordosis, sacral slope, and LIV sacrum MRi: Pfirrmann grading	Presence or not of LBP	There were differences in the occurrence of disc degeneration at MRI images between L3 and L4 groups were significant (p > .05) 45% of patients had low back pain at postoperative 10 years follow-up, but there was no correlation with disc degeneration or LIV No differences in average correction rates between L3 and L4 groups
Lavelle (2016)	Retrospective case series	120 (NR) 22 included	NR	NR	NR	Cotrel–Dubousset instrumentation	1 = L4 2 = L3 3 = L2 or above	NR	20 years (15 – 26)	QoL evaluation	NR	ODI, SF-36, VAS, SRS-22	Lowest instrumented segments did not show significant differences (not compared L3 to L4)
Ernecan (2016)	Retrospective case control-study	67 (10:57)	13.4 ± 1.78 (patients fused to L3) 13.7 ± 2.69 (patients fused to L4)	Lenke 1C: 6 Lenke 3C: 9 Lenke 4C: 8 Lenke 5: 5 Lenke 6: 7	Patients fused to L3: Lenke 1C: 4 Lenke 3C: 5 Lenke 4C: 4 Lenke 5: 6 Lenke 6:4 Patients fused to L4: Lenke 1C: 2 Lenke 3C: 4 Lenke 4C: 2 Lenke 5: 5 Lenke 6: 2	Pedicle screw instrumentation	Control group: 30 L3: 21 L4: 16	LIV determined based on central sacral vertical line on standing full-spine X-rays, concave supine bending film (L3 translation) and on-traction X-ray under general anesthesia. LIV was aimed to be horizontal and was checked intra-operatively after deformity correction	7.4 years (5–10, range)	MRi evaluation, QoL questionnaires And EOS full spine evaluation	MR = Pfirrmann grading for disc degeneration, Fujiwara grading for facet joint degeneration EOS = Cobb angle, coronal balance, thoracic kyphosis, lumbar lordosis, LIV tilt angle, LIV disc wedging angle	SRS-22, ODI, Numeric Rating Scale	There was no statistical difference for disc degeneration in all groups (p > .05). Clinical outcome scores were similar among all three groups (p > .05). Average correction rates in structural thoracolumbar/lumbar curve magnitudes were 78% in the L3 group and 79% in the L4 group, with no significant correction loss at the final follow-up (p > .05)
Akazawa (2017)	Retrospective case series	35 (5:30)	14.8	NR	NR	Harrington rod ± Wiring, Hooks, Zielkle method or Dwyer method	T12: 2 L1: 4 L2: 12 L3: 9 L4: 7 L5: 1 L group: L4 H group: L3	NR	35.1 years (27–45)	X-ray and MRi evaluation, QoL questionnaire	Disc degeneration: Pfirmann grading, and Modic changes X-ray: thoracic and lumbar Cobb angle, coronal balance and L3 and L4 vertebral tilt angles, Thoracic kyphosis (TK: T5-T12), thoracolumbar kyphosis (TLK: T10-L2), lumbar lordosis, pelvic incidence (PI), pelvic tilt (PT), and sagittal vertical axis	SRS-22, Roland-Morris Disability questionnaire, ODI	L4 group had significant advanced disc degeneration, less lumbar lordosis, greater SVA imbalance, and greater SVA compared to H group. No significant differences were seen in SRS-22, RDQ, and ODI among different groups
Akazawa (2018)	Retrospective case–control study	26 (4:22) 29 controls	14.8 (10–19)	22 thoracic curves 4 double major curves	NR	Harrington rod	T12:2 L1: 4 L2: 11 L3: 6 L4: 2 L5: 1	NR	36.1 (40–64)	MRi evaluation, X-ray evaluation, Qol Questionnaire SRS-22, ODI	Disc degeneration: Pfirmann grading, and Modic changes X-ray: Cobb, coronal balance, the tilt angle from the superior border of the vertebral body of the 1st to 5th lumbar spine, and the intervertebral wedging angle from the L1-2 to L5-S1. Thoracic kyphosis (TK: T5-T12 angle), thoracolumbar kyphosis (TLK: T10-L2), lumbar lordosis, pelvic incidence (PI), pelvic tilt (PT), and sagittal vertical axis	SRS-22, ODI	No significant differences in terms of disc\endplate degeneration in L3 LIV or L4 LIV SRS-22 scores for function and self-image were significantly lower in the AIS group ODI was significantly worse in the AIS group
Lonner (2018)	Retrospective case series	193 (27:166)	14.4	Lenke 1: 101 Lenke 2: 38 Lenke 3: 4 Lenke 4: 3 Lenke 5: 38 Lenke 6: 9	NR	Pedicle Screw instrumentation, hooks (only cited for LIV)	T8: 1 T9: 1 T10: 1 T11: 22 T12: 55 L1: 27 L2: 23 L3: 51 L4: 11 L5: 1	NR	10 years	X-ray evaluation, QoL evaluation	Disc degeneration measured at X-ray: A modified RMDD composite radiographic score (CRS) was calculated using the sum of each of the DD indicators: - Osteophytes - Schmorl’s nodes - Intradiscal calcifications - Sclerosis - Endplate shape X-ray:	SRS-22	LIV of L4 compared with more cephalad LIV had the highest risk of developing significant DD (27.3%; p < .0267). Moreover, a LIV translation of > 2 cm were 8 times more likely to develop significant disc degeneration than those with LIV translation ≤ 2 cm No significant association was established between 10-year CRS and SRS-22 scores
Chiu (2021)	Retrospective cross-sectional study	48 (3:45)	33.3 ± 8.7	NR	NR	Pedicle screw, hooks, wires instrumentation	T12: 1 L1: 1 L2: 5 L3: 12 L4: 27 L5: 2 G1: LIV L3 or higher G2: LIV L4 or lower	NR	17.7 ± 6.3	X-ray evaluation, MRi evaluation, QoL questionnaires	X-ray evaluation: Cobb, coronal balance, pelvic obliquity, Coronal LIV tilt, sagittal vertical axis, thoracic kyphosis, lumbar lordosis, pelvic tilt, sacral slope. Pelvic incidence, PI-LL mismatch Mri evaluation: Pfirmann grading	SRS-22, SF-36, ODI, Modified Cincinnati Sports Activity Scale (MCSAS)	Patients with fusion to L4 or lower had significant back pain. There were no significant differences in lumbar disc degeneration and the selection of LIV. There were no significant differences in the coronal and sagittal parameters between G1 and G2 groups except for LIV tilt (4.2° ± 8.8° in G1versus − 4.9° ± 12.0° in G2, p = 0.004). In the SRS-22r questionnaire, only pain domain demonstrated significant difference between G1 and G2 (4.3 ± 0.5 versus 4.0 ± 0.6, p = 0.044). Similarly, G1 obtained significantly higher scores in the bodily pain domain in SF-36 questionnaire, 88.7 ± 12.3 versus 77.8 ± 18.7 (p = 0.018). There were no significant differences in other domains of SRS-22r and SF-36 between both groups. There were also no significant differences in the ODI and MCSAS scores
Jakkepally (2022)	Retrospective case series	58 (6:52)	14.2 ± 2.4	Lenke 1: 22 Lenke 2: 2 Lenke 3: 26 Lenke 4: 3 Lenke 5: 5 Lenke 6: 0	NR	Pedicle screw instrumentation	T12: 1 L1: 12 L2: 2 L3: 13 L4: 28 L5: 2	LIV was selected based on stable, last touch, neutral and end vertebrae The lowest substantially touched vertebra was usually chosen as LIV	9.1 years	X-ray evaluation, MRi, QoL questionnaire	MRi Disc degeneration: Pfirmann grading + total end plate score (TEPS, derived from each disc by summing endplate defect scores of both rostral and caudal endplates of the disc. Patients, in whom the Pfirrmann's grade increased by one grade at the final follow-up in at least at one of the distal unfused levels, were classified under Pfirrmann's grade progression group (PGP). Those patients, in whom the disc degeneration had not progressed, were included in Pfirrmann grade static group (PGS) Facet degeneration: was measured in the unfused segments using Fujiwara's grading X-ray: Cobb angle, Apical vertebral translation, LIV tilt, LIV-Sacrum angle, sagittal and pelvic parameters	SRS-22 questionnaire	There was no correlation between higher grades of disc degeneration and lower instrumented vertebra (LIV) or functional outcomes scores (SRS-22) The progression of disc degeneration did not correlate with any X-ray parameters

*AIS* adolescent idiopathic scoliosis, *LBP* low back pain, *NR* not reported, *QoL* quality of life, *C7PL* C7 plumb line, *CSVL* central sacral vertical line, *ODI* Oswestry Disability Index

When studies involved both patients with LIV selection not limited solely to L3–L4, data about patients with L3 or L4 as LIV selection were pooled: if this was not possible, the study was excluded.

### Methodological quality assessment of included studies

**Fig. 3:**
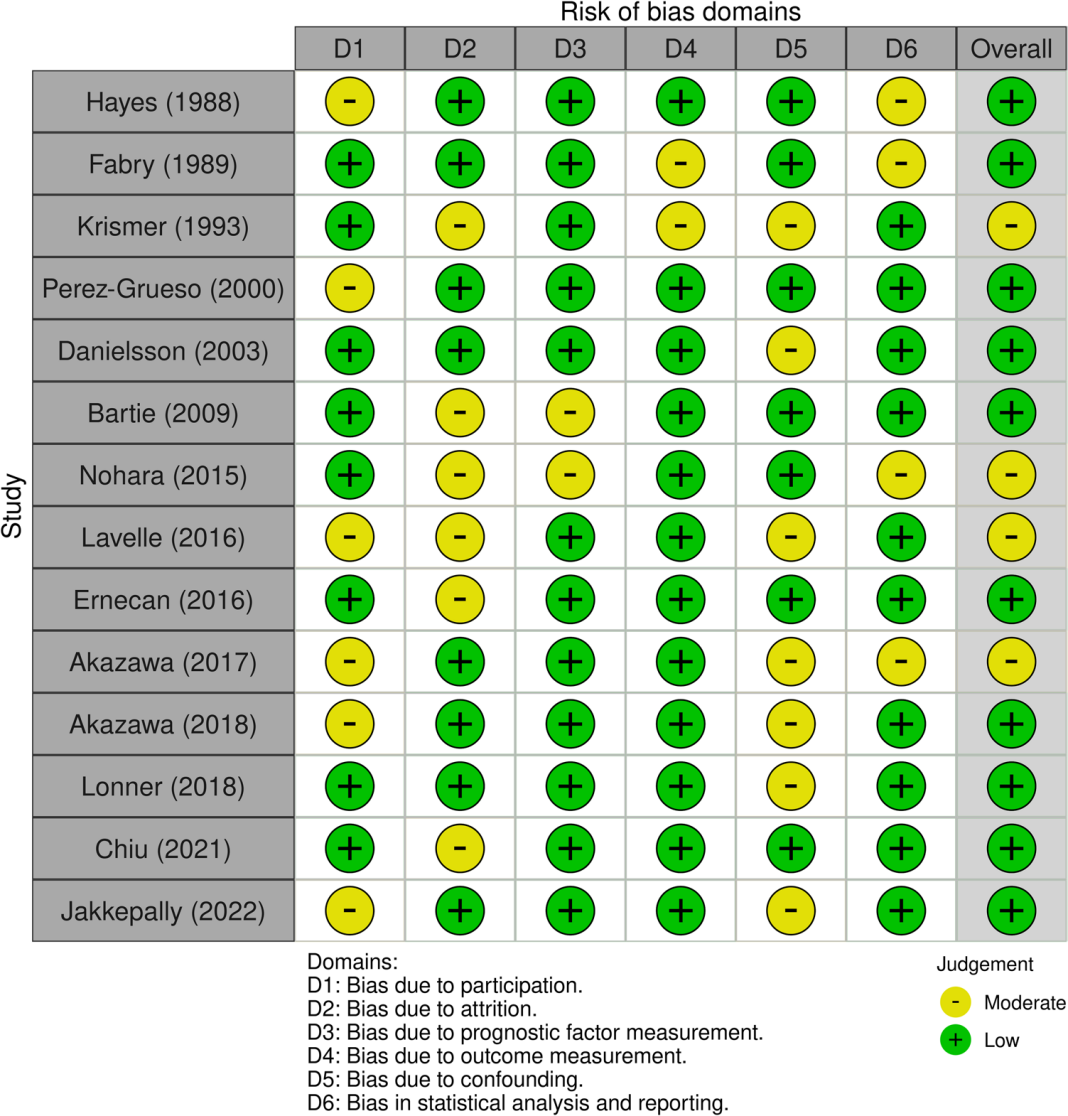
Risk of bias assessment of the included study in qualitative synthesis according to QUIPS tool

**Fig. 4:**
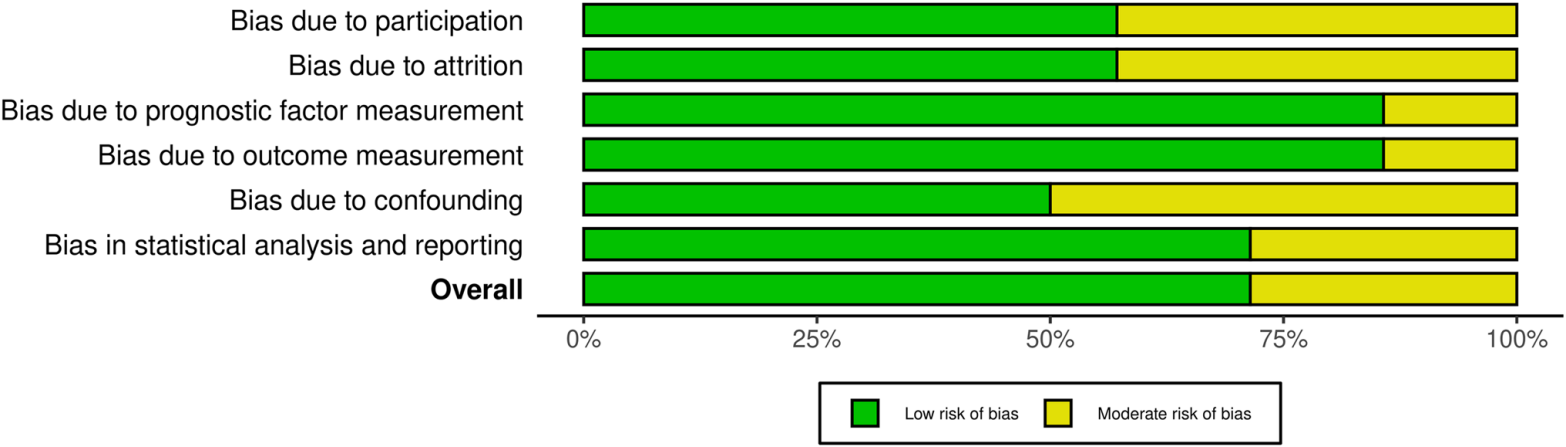
Weighted bar plots of the distribution of risk-of-bias judgments within each bias domain

The Quality in Prognosis Studies (QUIPS) tool[18] was used to assess the methodological quality of the included studies. The quality of each study was reported assessing 6 domains: study participation, study attrition, prognostic factor measurement, outcome measurement, study confounding, and statistical analysis and reporting. Each domain can present a low, moderate, or high risk of bias: these combined together form an overall risk of bias. For each included study, the total risk of bias was categorized as low risk with ≥ 4 low-risk domains, moderate risk with < 4 low-risk domains, and high risk with ≥ 1 high-risk domains. As with the evaluation of titles and abstracts, any disagreement was solved by the senior Author (CF). Details on the quality of the studies included are summarized in Figs. 3 and 4.

### Statistical analysis

Meta-analyses were performed when possible. Heterogeneity between studies was assessed using the inconsistency statistic (I^2^ > 75% was considered as highly heterogeneity). Publication bias was assessed with Egger’s test and represented with forest plots. Odds ratio with 95% CI and *p* value were used as a measure of effect size. A random-effects model was applied. All statistical analyses were conducted with Jamovi version 2.2 (The Jamovi Project, Sydney, Australia) software. *p* value < 0.05 was considered to be significant.

### Informed consent and institutional review board approval

Ethical approval and institutional review board approval were not required, because this study would retrieve and synthesize data from already published studies.

## Results

### Included studies

A total of 257 studies were found through electronic search. After screening, 14 studies [19–32] met the inclusion criteria and were included in the systematic review. There were eight [19, 20, 22, 24–26, 30, 32] retrospective case series, four [21, 27, 28, 31] retrospective case–control studies, one [23] retrospective cross-sectional study, and one [29] retrospective cohort study.

In all analyzed studies, each group of patients in which L3 was selected as LIV was matched with a relatively homogeneous group composed by patients undergoing similar surgery with L4 as LIV.

Included studies analyzed both small and large-sized populations, describing the clinical and radiological outcomes in surgically treated adolescent idiopathic scoliosis patients in whom L3 or L4 were selected as LIV. Included studies are heterogeneous in the description of curve pattern/Lenke type, radiological assessment, clinical assessment, and population demographics (Table 1).

### Risk-of-bias assessment

Two authors (MM and MT) assessed the risk of bias for each study using the QUIPS tool, and results are shown in Fig. 3 and 4.

All studies [19–32] indicated an overall risk of bias low or moderate (respectively 73 and 27%). Since most studies well described the outcome measurement with a clear definition of the result, accurate and reliable outcome measurements, and outcome assessment, they demonstrated a low outcome and prognostic factor measurement items (< 85%). Furthermore, the confounding measurement and account item was consistently moderate for half of the studies since the observed influence of prognostic variables on outcome may be skewed by another component linked to the outcome. The other half of the studies performed multiple multivariate analysis to reduce the influence of confounding variables and identify independent risk factors for disc degeneration.

### Cohort demographics

Included studies reported data on a total of 1264 patients (1004 females, 79.4%) who underwent radiological and/or clinical assessment at long-term follow-up after adolescent idiopathic scoliosis surgery. The median age at first evaluation ranged from 13.4 ± 1.78 to 33.3 ± 8.7 years and the mean follow-up ranged from 7.4 to 36.1 years, with a mean of 15.9 years.

### Curve pattern and type of instrumentation

Only four studies [22, 24, 29, 31] reported data regarding the Lenke pattern of scoliotic curves (Table 1). The other studies reported various patterns of scoliotic curves (e.g., thoracic, lumbar, and double), because, in most cases, patients were treated before the introduction of the Lenke classification. In the cohort reported by Nohara et al. [29], patients were classified according to Lenke retrospectively.

Regarding the type of instrumentation, the included studies utilized various techniques and approaches. Eight studies employed Harrington rod [19–21, 25–28, 32], while 5 studies utilized all pedicle screws and rods [22, 24, 29, 31]. Only one study [30] reported the use of Cotrel–Dubousset instrumentation. Remnant studies reported the use of hybrid instrumentations (e.g., wires and hooks) either segmental or non-segmental [22, 23, 29, 32].

### LIV assessment

Twelve studies reported the LIV selection of the included patients [20–26, 28–32] (Table 1). Two studies [19, 27] reported LIV as selection of either high or low lumbar vertebrae (such as L2 or above/L3 or below). The included studies reported data on a total of 116 thoracic LIVs, 200 L1 LIV, 301 L2 LIV, 342 L3 LIV, 332 L4 LIV, 89 L5 LIV, and 4 S1 LIV. The majority of included studies did not clearly define LIV selection criteria, and details regarding LIV selection criteria are depicted in Table 1.

### X-ray assessment

All the included studies, except two [20, 30], evaluated their cohort with an X-ray study. The X-ray evaluation focused on the most widely recognized radiographic indexes of scoliosis preoperative planning, such as Cobb angle, apical vertebral rotation and translation, coronal and sagittal alignment, thoracic kyphosis, lumbar lordosis, sacral slope, pelvic tilt, and PI-LL mismatch. LIV radiographic parameters were evaluated in seven studies [22–24, 28, 29, 31, 32], measuring the LIV tilt, LIV disc angle, and LIV-to-S1 lordosis. Three [19, 25, 26] studies evaluated the LIV anteroposterior translation motion on lateral flexion and extension lumbar spine X-ray, to detect possible instabilities and/or listhesis.

Hayes et al. [19] in their retrospective case series of 48 AIS patients surgically treated with Harrington instrumentation described an increased incidence of retrolisthesis and translational motion at junctional level in patients fused to L4 when compared with patients fused to L3 or above (13/16, 81%, retrolisthesis when fused to L4 vs 4/10, 40%, when fused to L3; 4.4 mm of average translational motion when fused to L4 vs 3.5 mm when fused to L3).

Analogous results were obtained by Perez-Grueso et al. [26], where a higher rate of anteroposterior translation at junctional level was found between patients fused to L4 rather than L3. In 10 of 32 patients, the first disc below fusion showed instability at flexion–extension radiographs. However, those findings did not show significant differences among groups and were described by the authors as similar in degree and incidence to those found in an asymptomatic aging population.

Akazawa et al. [32] evaluated the lumbar disc degeneration among two groups of surgically treated AIS patients, L group where LIV was L4 or below and H group where LIV was L3 or above. The authors found that L group showed less lumbar lordosis than the H group (48.1° vs 32.2°) and greater SVA (1.2 vs 5.5 cm).

Lonner et al.[22] conducted a comprehensive evaluation of disc degeneration indicators at radiographic imaging, including disc space height, presence of osteophytes, Schmorl's nodes, intradiscal calcification, and endplate sclerosis or shape modifications. Through a composite radiographic score (CRS), calculated by summing each indicator, they analyzed the eventual association between degenerative changes at X-rays and LIV selection. The findings showed that patients fused to L4 exhibited a higher risk of significant disc degeneration (27.3%; *p* < 0.0267) compared to those with more cephalad LIV. Moreover, patients with LIV translation > 2 cm from the central sacral vertical line (CSVL) were eight times more likely to develop significant disc degeneration than those with LIV translation ≤ 2 cm and patients fused caudal to L3 were five times more likely to develop significant disc degeneration 10 years after surgery. No significant influence of LIV tilt or disc wedging on disc degeneration was found.

The other included studies [21, 23–25, 27–29, 31] did not show any statistically significant differences between radiological parameters and LIV choice in surgically treated AIS patients.

### MRI evaluation

Six included studies[23, 24, 26, 29, 31, 32] performed MRI evaluation at long term-follow-up. Most of the studies performed MRI imaging at last follow-up evaluation searching for degenerative changes in unfused distal levels. Jakkepally et al. [24] compared pre-operative and post-operative MRI images looking for degenerative disc disease progression. The data were extracted to assess degenerative disc disease rates in patients in whom spinal fusion was extended to L3 or L4. Results of the studies regarding MRI evaluation are summarized in Table 2.

**Table 2:** Characteristics of included studies that evaluated magnetic resonance imaging at long-term follow-up and pooled data regarding patients in which LIV selection was L3 or L4

Authors	Patients (n, f/m)	Mean patients age at surgery (years)	Curve patterns	LIV L3 (n)	LIV L4 (n)	Disc degeneration criteria	DD + LIV L3 (n, %)	DD + LIV L4 (n, %)	Follow up (years)	Pre-operative Cobb angle of lumbar/TL curve	Pre-operative LL	Pre-operative LIV tilt	Last FU Cobb angle of lumbar/TL curve	Last FU LL	Last FU LIV tilt	Spinal instrumentation
Jakkepally 2022	58	14.2 ± 2.4 (DD-) 13.9 ± 2.5 (DD +)	Lenke types: 1:22 2:2 3: 26 4:3 5:5	13	28	Patients in whom the Pfirrmann’s grade increased by one grade at final follow-up in at least one of the distal unfused levels, were classified under Pfirrmann progression group (DD +)	3 (23.1%)	8 (28.6%)	9.1 ± 0.6 (DD-) 9.2 ± 0.7 (DD +)	46.2 ± 15.3 (DD-) 42.2 ± 13.0 (DD +)	40.0 ± 8.4 (DD-) 39.6 ± 8.4 (DD +)	17.9 ± 5.4 (DD-) 19.0 ± 7.4 (DD +)	16.2 ± 9.1 (DD-) 16.2 ± 10.2 (DD +)	40.1 ± 6.3 (DD-) 42.3 ± 5.9 (DD +)	4.2 ± 3.5 (DD-) 4.9 ± 3.1 (DD +)	All pedicle screws construct
Chiu 2021	48 (3/45)	10—18	/	12	27	Pfirrmann grades > 3 were considered disc degeneration + (no pre-operative MRI)	7 (58.3%)	11 (40.7%)	17.7 ± 6.3	/	/	/	/	48.1 ± 15.0	- 1.3 ± 11.7	Hybrid instrumentation (posterior pedicle screws, hooks, wires)
Akazawa 2017	16 (2/14)	14.7 ± 2.0	/	9	7	Pfirrmann grades > 3 were considered disc degeneration + (no pre-operative MRI)	5 (55.6%)	7 (100%)	35.1 (27–45)	/	/	/	26.7 ± 9.9	43.3 ± 17.0	8.3 ± 7.0	Anterior or posterior instrumentation (Harrington instrumentation, Zielke, Dwyer, Luque, Hybrid)
Enercan 2016	37 (7/38)	13.4 ± 1.8 (LIV = L3) 13.7 ± 2.7 (LIV = L4)	Lenke types: 1C: 6 3C: 9 4C: 8 5: 5 6: 7	21	16	/	/	/	LIV = L3: 7.4 (5 – 10) LIV = L4: 9.0 (5 – 17)	LIV = L3: 41.0 ± 11.0 LIV = L4: 54 ± 8	LIV = L3: - 30 (-18 – -45) LIV = L4: - 35	/	LIV = L3: LIV = L4:	LIV = L3: LIV = L4:	LIV = L3: 3.7 (0–12) LIV = L4: 5.1 (0–12)	All pedicle screws construct
Nohara 2015	93 (3/90)	15.2 (11 – 20)	Lenke types: 1: 58 2: 23 3: 4 5: 3 6: 5	28	2	Pfirrmann grades > 3 were considered disc degeneration + (no pre-operative MRI)	19 (68%)	1 (50%)	12.8 (10.0 – 17.9)	39.4 ± 12.9	- 55.1 ± 11.4	21.0 ± 8.1	16.9 ± 8.0	- 53.1 ± 15.2	6.8 ± 4.0	Hybrid instrumentation (posterior pedicle screws with or without screws)
Perez-Grueso 2000	35 (2/33)	15 (11 – 25)	26 double major, 2 lumbar, 6 TL, 1 thoracic	13	17	/	/	/	12.0 (10.0 – 22.0)	51.9 ± 11.2	- 57.3 ± 12.8	22.0 ± 5.5	25.3 ± 10.0	- 51.2 ± 9.3	10.4 ± 5.7	Cotrel–Dubousset instrumentation

**Fig. 5:**
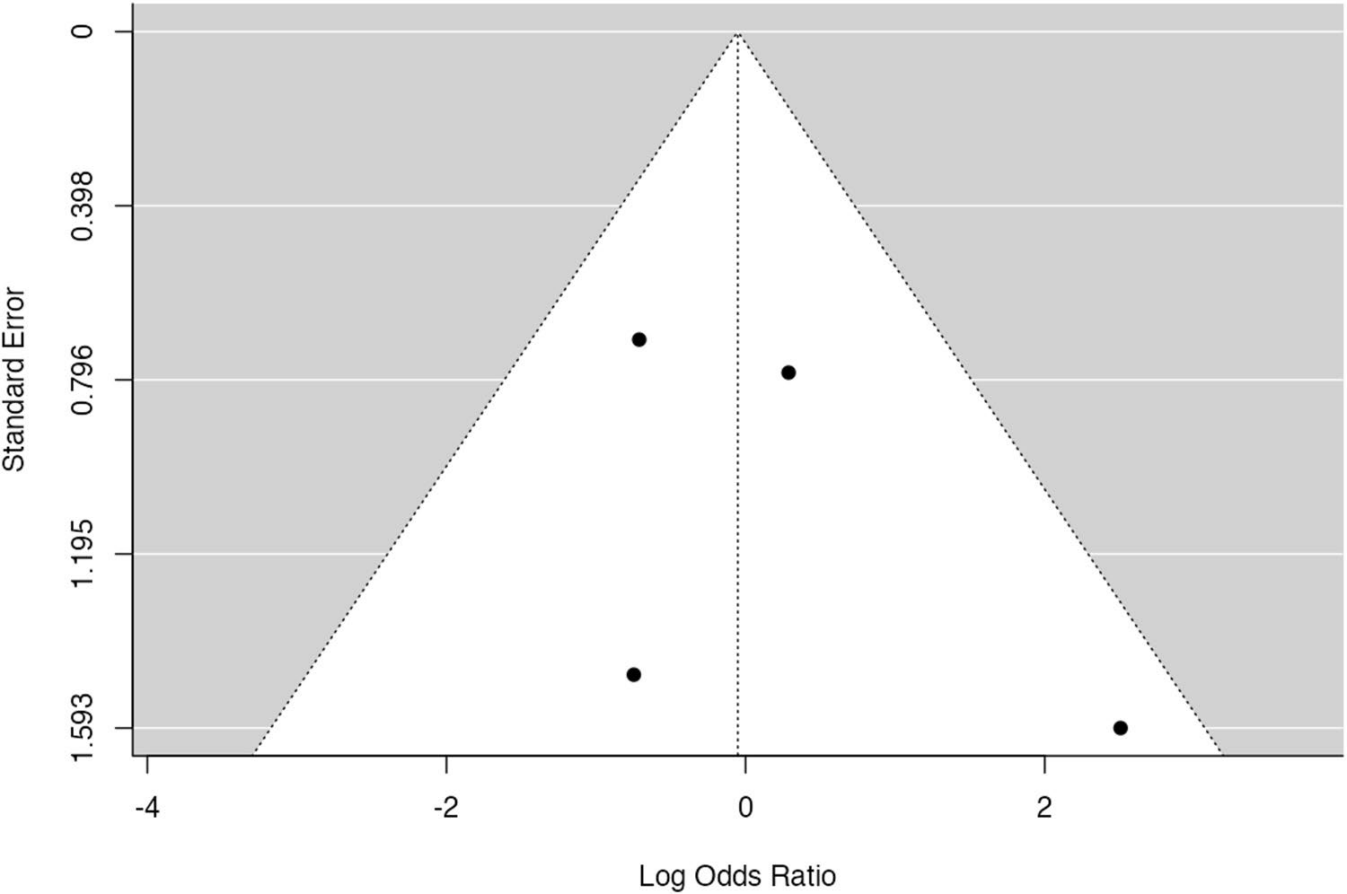
Funnel plot of effect sizes for publication bias of the included study in meta-analysis

**Fig. 6:**
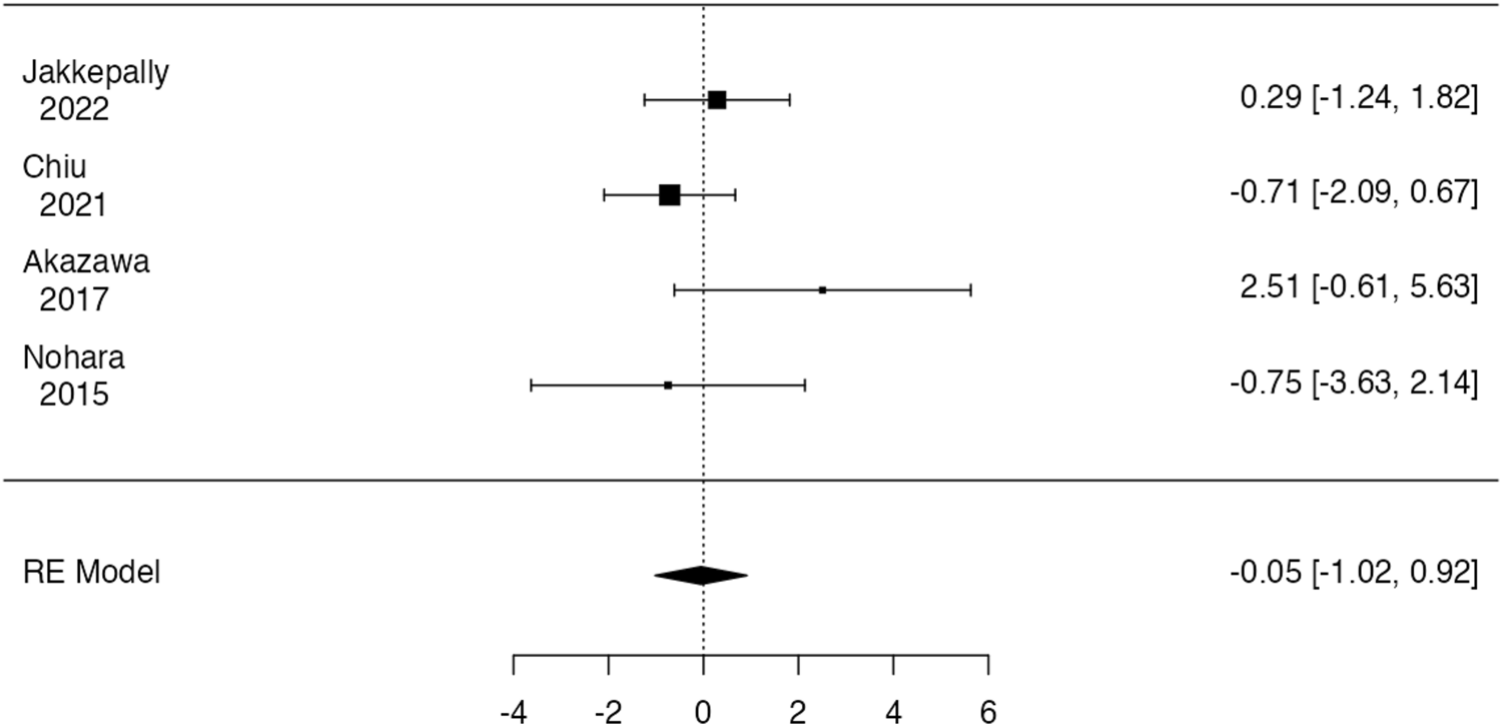
Forest plot of overall meta‐analysis of the included studies with data about disc degeneration rate observed at MRI images at long-term follow-up in patients in

Meta-analysis was conducted on comparative studies and studies in whom data regarding patients with LIV selection of L3 or L4 could be pooled. No significative heterogeneity (*I*^2^ = 5.88, *p* = 0.276) and publication bias (*p* = 0.286, Fig. 5) were found. No significant difference in disc degeneration, intended as Pfirrmann’s grade progression or Pfirrmann’s grade > 3 in at least one intervertebral disc distal to fused region at last follow-up, between patients fused at L3 or L4 was found at meta-analysis (*p* = 0.916, Fig. 6), with an estimated average log odds ratio of – 0.05 (95% CI −1.019 to 0.915).

Perez-Grueso et al. [26] found that 60% of evaluated intervertebral discs in distal unfused segments showed degenerative changes at more than 10 years of follow-up. Those findings did not correlate with number of unfused segments, radiographic results of curve correction, and final clinical outcome. The authors did not provide data regarding degenerative disc disease classification.

Nohara et al. [29], Akazawa et al. [32], and Chiu et al. [23] evaluated MRI images at last follow-up and found significant degenerative changes in the unfused distal lumbar segments. However, pre-operative MRI images were not available. Nohara et al. [29] found that degenerative disc disease (Pfirrmann grade > 3) was present in at least one unfused disc in 68 and 50% of patients fused down to L3 and L4, respectively. It is important to notice that only 2 patients in the cohort had been fused to L4. Akazawa found that 100% of patients in which L4 was chosen as LIV showed degenerated disc below fusion, on the contrary only 55% of patients fused to L3 showed disc degeneration. Chiu et al. [23] found higher rate of disc degeneration in patients fused to L3, rather than and L4, 58.3 and 40.7%, respectively. Jakkepally et al. [24] evaluated the eventual progression of disc degeneration comparing pre-operative MRI images with MRI images obtained at last follow-up (mean follow-up of 9.1 years). Eleven patients showed degenerative progression from Pfirrmann’s grade 1 to grade 2. Only in 4 patients, Pfirrmann’s grade progressed to value higher than 3, which is commonly considered the cut-off to define a degenerated disc. No patient progressed to Pfirrmann’s grade 5.

### Clinical assessment

Clinical assessment of included patients was performed in all studies [19–32]. Patient reported outcomes measures (PROMs) represented the used tools to measure patient-reported outcomes in most of the included studies. The following tools were administered: SRS-22 [33] with all its domains (Function, Pain, Self-Image, Mental Health, and Satisfaction with management), SF-36 [34] (short form health survey), ODI [35] (Oswestry Disability Index), VAS [35] (Visual analogue scale), NRS [35] (Numeric rating scale), Roland-Morris Disability questionnaire [35], and Modified Cincinnati Sports Activity Scale (MCSAS) [35].

Other studies focused their clinical evaluation in assessing the presence/absence of low back pain or using questionnaires or classifications such as Moskovitz, Moe, Winter and Binner low back pain classification [19], Arbitrary Scale of Back pain and activity Grading [20], Dallas pain questionnaire [25], and Ransford pain drawings [25].

Hayes et al. [19] investigated the presence of low back pain in 48 surgically treated AIS patients with a mean follow-up of 11 years. Patients fused to L4 when compared with patients fused to higher levels showed higher incidence of low back pain, particularly in those who showed retrolisthesis in dynamic lumbo-sacral X-ray (77% of those showing retrolisthesis). These results were confirmed by Fabry et al.’s [20] study, where postoperative severe low back pain increased from patients fused to L3 (17%) to patients fused to L4 (37%), concluding that ideally the LIV should not go beyond L2, and lower fusions are prone to give more low back pain. In Krismer et al.’s [25] study, low back pain depended on fusion level, with occurrence of 80% of pain in patients fused down to L4. Nevertheless, no relationship between LIV or pain on the one hand and segmental range of motion or increased translation on the other hand, and none between lordosis, pain and increased translation was found. Chiu et al. [23] divided their population study in Group 1 (G1) and Group 2 (G2) depending on LIV selection, L3 or higher and L4 or below, respectively. They found that in the SRS-22r questionnaire results, only pain domain demonstrated significant difference between G1 and G2 (4.3 ± 0.5 versus 4.0 ± 0.6, *p* = 0.044). Similarly, G1 obtained significantly higher scores in the bodily pain domain in SF-36 questionnaire (88.7 ± 12.3 versus 77.8 ± 18.7 (*p* = 0.018)). The other included studies [21, 24–32] did not show any statically significant differences between clinical parameters\PROMs results and LIV selection in surgically treated AIS patients. Table 3 reports PROMs of included studies stratified per LIV selection.

**Table 3:** Patient-reported outcomes measures details of the included studies, stratified per LIV selection

First author (Year)	N° Patients (M:F)	N° LIV L3	N° LIV L4	PROMs L3	PROMs L4	Mean follow-up	Spinal instrumentation
Hayes (1988)	48 AIS patients (3:45) 59 Controls	NR	NR	NR	NR	11 years (range, 1–23)	Harrington rod
Fabry (1989)	182 (44:138)	34	32	Grade 1:14 Grade 2:10 Grade 3:4 Grade 4:4 Grade 5:2 Grade 6:0	Grade 1:6 Grade 2:10 Grade 3:4 Grade 4:10 Grade 5:2 Grade 6:0	7 years (range, 4–14)	Harrington rod
Krismer (1993)	49 (7:42)	6	9	NR	NR	14 years (range 11–22)	Harrington rod
Perez-Grueso (2000)	35 (2:35)	15	18	NR	NR	10 years	Harrington rod
Danielsson (2003)	139 AIS patients (10:129) 100 controls (10:90)	NR	NR	NR	NR	23 years	Harrington rod
Bartie (2009)	171 AIS patients (18:153) vs 209 healthy controls	NR	NR	NR	NR	10 years	Harrington rod
Nohara (2015)	93 (3:90)	28	2	NR	NR	12,8 years (10–17)	Hybrid instrumentation (screws, hooks, wires)
Lavelle (2016)	22 AIS patients	5	3	Median values VAS-Back Pain 1 SRS-22 Total 4.08 ODI 8 Mean Values VAS-Back Pain 2.2 SRS-22 Total 3.97 ODI 17.2	Median values VAS-Back Pain 2 SRS-22 Total 4.06 ODI 8 Mean Values VAS-Back Pain 2 SRS-22 Total 4.22 ODI 9.33	20 years (15 – 26)	Cotrel–Dubousset instrumentation
Ernecan (2016)	67 (10:57)	21	16	Median values SRS-22r Pain: 4.5 Self-Image: 4.1 Function: 4.6 Mental Health: 3.9 Satisfaction: 4.5 Subtotal: 4.3 ODI: 5.6 NRS Back pain: 1.5 Leg Pain: 0.4	Median values SRS-22r Pain: 4.6 Self-Image: 4.3 Function: 4.7 Mental Health: 3.9 Satisfaction: 4.8 Subtotal: 4.5 ODI: 4.0 NRS Back pain: 1.1 Leg Pain: 0.2	7.4 years (5–10, range)	Pedicle screw instrumentation
Akazawa (2017)	35 (5:30)	27	8	Mean values SRS-22r Pain: 4.5 Self-Image: 3.0 Function: 4.3 Mental Health: 4.2 Satisfaction: 3.7 ODI: 6.8	Mean values SRS-22r Pain: 4.3 Self-Image: 2.9 Function: 4.4 Mental Health: 4.0 Satisfaction: 3.3 ODI: 8.9	35.1 years (27–45)	Harrington rod ± Wiring, Hooks, Zielkle method or Dwyer method
Akazawa (2018)	26 (4:22) 29 controls	6	2	NR	NR	36.1 (40–64)	Harrington rod
Lonner (2018)	193 (27:166)	51	11	NR	NR	10 years	Pedicle Screw instrumentation, hooks (only cited for LIV)
Chiu (2021)	48 (3:45)	19	29	Mean values SRS-22r Pain: 4.3 Self-Image: 3.6 Function: 4.1 Mental Health: 4.1 Satisfaction: 4.2 Subtotal: 4.1 ODI: 8.9 SF-36: 81.9	Mean values SRS-22r Pain: 4.0 Self-Image: 3.8 Function: 4.1 Mental Health: 4.1 Satisfaction: 4.0 Subtotal: 4.0 ODI: 12.9 SF-36: 75.6	17.7 ± 6.3	Pedicle screw, hooks, wires instrumentation
Jakkepally (2022)	58 (6:52)	13	28	NR	NR	9.1 years	Pedicle screw instrumentation

*NR* not reported

## Discussion

The surgical management of adolescent idiopathic scoliosis has undergone substantial advancements in recent decades, driven by both technological developments in instrumentation and conceptual breakthroughs leading to new classification systems that better stratify patients and guide surgical treatment. A milestone in pre-operative planning was reached with the introduction of the concept of "structural curve" which allowed to clearly define criteria for ideal fusion area and identified compensatory/non-structural curves that might undergo spontaneous correction after surgery. According to Lenke classification [1], inclusion of all structural curves in the spinal fusion area is recommended. Thus, when dealing with double major or TL/L curves (Lenke pattern 3, 4, 5 and 6), the fusion area must be extended to the lumbar spine, and the usual recommendation for the selection of the lowest instrumented vertebra (LIV) falls on L3 or L4 based on TL/L factors [1, 3, 10].

Up to date, spine surgeons still face the concern of whether to spare a distal lumbar segment, accepting a higher risk of postoperative coronal imbalance [36], greater LIV–S1 coronal Cobb angle and suboptimal LIV tilt, or to achieve better coronal correction and LIV leveling at the cost of sacrificing a motion segment. There is a moderate consensus on the importance of leveling the LIV using distraction and/or compression, allowing further improvement of the residual lumbar curve over time [10]. The long-term outcomes of preserving a distal motion segment remain unknown, since there is no long-term study that clarifies whether there is a clinical and radiological benefit from leaving an unfused lumbar spine with moderate residual curvature over a partially fused lumbar spine with leveled subadjacent disk spaces [10].

A few studies have examined the effects of long spinal fusions performed in adolescent patients on unfused caudal segments using magnetic resonance imaging (MRI).

When considering all the eligible studies, the overall meta-analysis showed that the LIV selection of L3 rather than L4 in adolescent idiopathic scoliosis surgery does not lead to a significant difference in disc degeneration rate at MRI evaluation in distal unfused lumbar segments at long-term follow-up (*p* = 0.916).

A possible explanation of the findings of the present work is that DD in regions not included in the spinal fusion may proceed as acceleration of physiological aging processes, affecting particularly the junctional and lumbosacral region which are subjected to higher stresses. Consequently, a certain degree of degenerative acceleration occurs after each spinal fusion surgery, and this acceleration may depend on the overall extension of the fusion area. As a result, addition of a distal level may represent a relatively modest increase in lever arm and junctional stresses, including those at the lumbosacral junction. Additionally, the relative effect of the residual lumbar curve is still unclear, so the weight of an addition of one distal level to the spinal fusion and a greater LIV–S1 coronal Cobb angle might balance each other with the result of a similar disc degeneration rate at long term.

Two distinct pathological conditions can be distinguished based on the location of disc degeneration. The first is adjacent segment disease (ASD) in which the affected disc is placed at junctional level and clearly subjected to overload and hypermotility. ASD is defined as new degenerative changes at a level adjacent to a spinal fusion, accompanied by related symptoms (radiculopathy, myelopathy, or instability). This condition typically occurs within a short time span from index surgery, making it often detectable during the conventional 2-year follow-up in cohorts of adult patients who underwent spine fusion [37, 38].

The above-mentioned condition differs from the acceleration of physiological disc degeneration processes that also occur in healthy individuals over long time span and affects all unfused discs, but predominantly the lumbosacral junction, with L5-S1 being the most affected level which is naturally more vulnerable due to the frequent concomitance of AIS with low pelvic incidence morphotypes [26, 39].

Another important factor to consider is the clinical impact of disc degeneration. In the cohort of Green et al., healthy asymptomatic patients showed a prevalence of disc degeneration approximately 38%, with the majority of affected discs being at the L5-S1 level. However, the occurrence of a similar degree of degeneration is found in 100% of patients who have undergone long spinal fusion, with 45% of patients having a Pfirrmann grade > 3 at over 10 years of follow-up. The most affected level was again L5-S1, with only one patient showing significant adjacent segment disc degeneration.

Clinical assessments of patients of the included studies at long-term follow-up show a slight tendency to have worse clinical outcomes over the long term in terms of back pain and PROMs if spinal fusion is extended to L4 rather than L3. Despite that, meta-analysis could not be performed on clinical parameters because of heterogeneity of evaluated PROMs in included studies.

Fabry et al. [20] reported 17% back pain in patients fused to L3, 37% in patients fused to L4 and 46% in patients fused to L5. On the contrary, Enercan et al. [31] reported no significant difference in any of SRS domain between patients fused to L3, patients fused to L4 and control group. Bartie et al. [28], Ding et al. and Danielsson et al. [27, 40] found no statistically significant relationship between LIV and any of the evaluated clinical parameters; furthermore, no measurement was found to be predictive of a painful outcome. Patients with L4 as LIV showed a tendency to have a more intense pain, but not a more frequent or durable back pain. Up to date, the clinical implications of preserving one motion segment below a long spinal fusion extended to the lumbar spine is still uncertain.

**Fig. 7:**
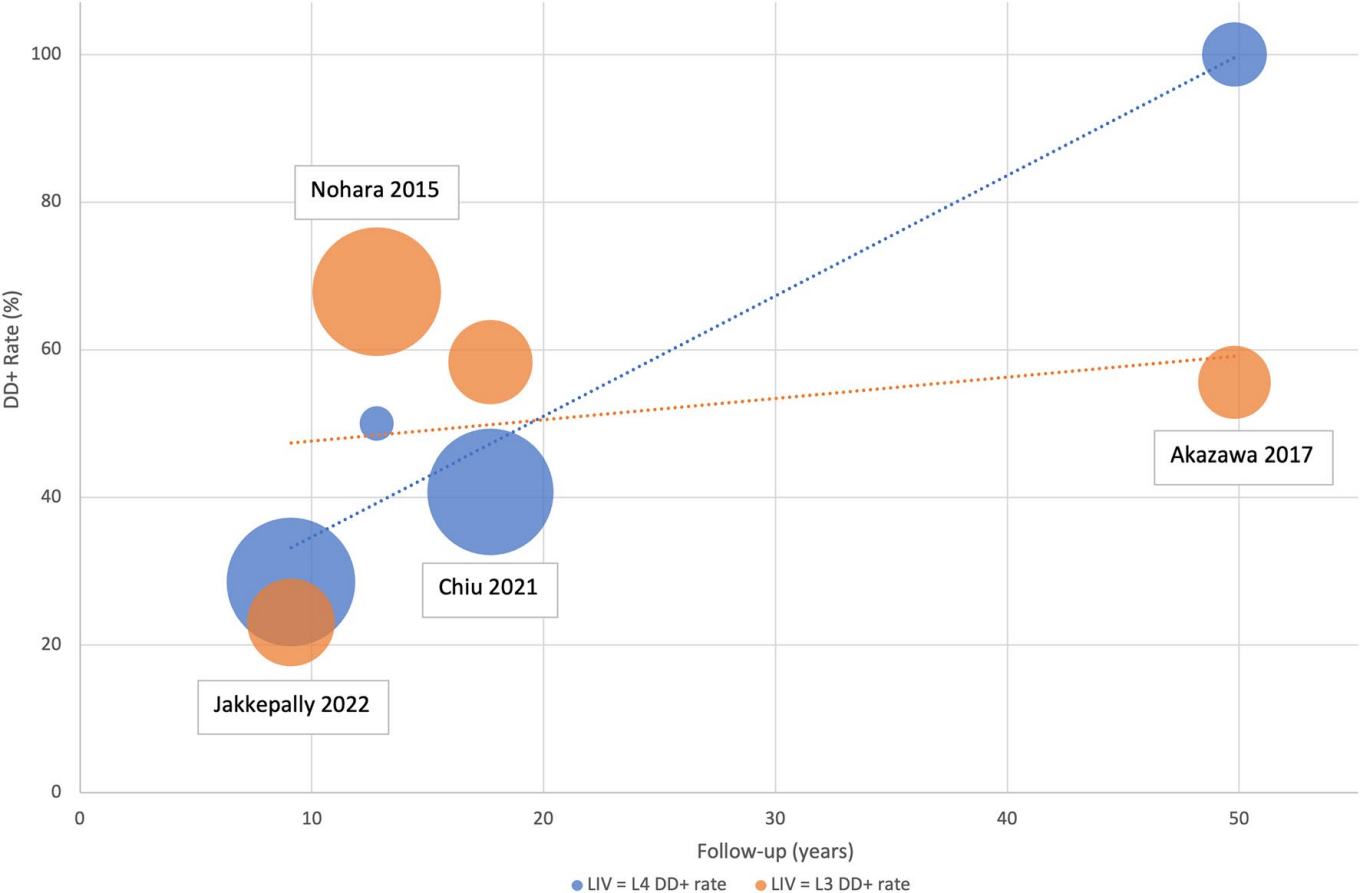
Graph depicting the disc degeneration rate over different follow-up periods in patients of the included studies, based on whether L3 or L4 was selected as LIV

The present work does not come without limitations; the heterogeneity of follow-up periods among papers is one of the major confounding features, since development of degenerative disc disease is a strictly time-dependent phenomenon, 10 years of time might be a short time to observe disc degeneration and to establish any cause-effect relationship between spinal fusion and DDD, since disc degeneration also occurs in healthy patients starting from second–third decade of life [41]. Based on these assumptions, true outcomes can solely be appraised through prolonged patient follow-up over an extended period, allowing the disc degeneration acceleration resulting from spinal fusion to become more evident, as demonstrated in Fig. 7 [42].

Moreover, it is important to consider the post-operative sagittal spinal alignment of instrumented spine. Different spinal instrumentations determine different post-operative sagittal alignments. Included studies were heterogeneous in term of surgical techniques (anterior or posterior instrumentation) and spinal instrumentations (Harrington rod, Cotrel–Dubousset instrumentation, Hybrid instrumentation, Pedicle screws, and rod). Jakkepally et al. [24] included patients treated only with posterior spinal fusion with pedicle screws and rods, Chiu et al. [23] included patients treated with both segmental and non-segmental instrumentation. Akazawa et al. [32] included patients treated with both anterior and posterior instrumentation. Perez-Grueso et al. [26] included patients treated with Cotrel–Dubousset instrumentation.

Harrington instrumentations extended in the lower lumbar spine often determines lumbar flatback and subsequent compensatory hyperlordosis of distal unfused segments leading to degenerative changes such as retrolisthesis, facet joint arthrosis, spinal stenosis, and DDD [43]. In fact, patients treated with Harrington instrumentation often exhibit a higher rate of degenerative disc disease compared to those treated with segmental instrumentation, which enables surgeons to achieve a more physiological sagittal alignment. Akazawa et al. [32] reported 100 and 45% of disc degeneration in patients fused to L4 and L3, respectively. On the contrary, Jakkepally et al. [24] reported significantly lower rate of disc degeneration with 28.6 and 23.1% of disc degeneration rate in patients fused to L4 and L3, respectively.

Another confounding factor is that the clinical impact of disc degeneration (Pfirmann grade > 3 or Pfirmann grade progression over time) might be modest in some patients, as similar degenerative changes can be observed in a relatively high percentage of asymptomatic subjects [44].

An additional limitation of the study is attributed to the general poor quantity and quality of the literature available. In the majority of cases, the endpoint of these works was the qualitative assessment of the long-term degeneration status of the spine following spinal fusion from both clinical and radiographic point of view.

Another major confounding factor of the present work may be represented by the lack of information in included studies regarding LIV selection criteria and comparability assessment of curves fused to L3 rather than L4.

Finally, included studies that evaluated clinical parameters were heterogeneous in terms of PROMs’ assessment and cut-off criteria for DDD definition.

## Conclusion

A few studies previously suggested that the LIV selection of L3 or L4 may not have an impact on disc degeneration over the long term both from the clinical and radiological point of view. This review, although the numerous limitations, confirmed the absence of significant clinical and radiological differences between patients fused to L3 or L4. Long-term studies of patients treated with contemporary spinal instrumentations are needed.

### Supplementary Information

Below is the link to the electronic supplementary material.

Supplementary file1 (DOCX 17 KB)

## Data Availability

Not applicable.
