# Supplementary material for: Comparable rates of lumbar disc degeneration at long-term following adolescent idiopathic scoliosis spinal fusion extended to L3 or L4: systematic review and meta-analysis
Source: Spine Deform. 2024 Mar 28;12(4):877–901. doi: 10.1007/s43390-024-00849-4 (PMC11217089; doi:10.1007/s43390-024-00849-4)
Supplement: Supplementary file 1 — Supplementary file1 (DOCX 17 KB) [file 43390_2024_849_MOESM1_ESM.docx]

|  | **Concept 1** | **Concept 2** | **Concept 3** | **Concept 4** |
| --- | --- | --- | --- | --- |
| **Key concepts** | Adolescent Idiopathic Scoliosis | Lower Instrumented Vertebrae | Disc Degeneration | Outcomes |
| **Free text terms / natural language terms**  (Synonyms, UK/US terminology, medical/laymen’s terms, acronyms/abbreviations, drug brands, more narrow search terms) | Scoliosis, idiopathic scoliosis, adolescent scoliosis | Arthrodesis Area,  Last lumbar vertebra | Discopathy, lumbar discopathy, degenerative disc disease | PROMS, adjacent segment degeneration |
| **Subject fields**  (Title, abstract, keyword) | Title, abstract, keyword | Title, abstract, keyword | Title, abstract, keyword | Title, abstract, keyword |

**Table S1:** Search strategy table
